# 5,7-Dimeth­oxy-2-(4-methoxy­phen­yl)-4*H*-1-benzopyran-4-one methanol solvate monohydrate

**DOI:** 10.1107/S1600536809040513

**Published:** 2009-10-10

**Authors:** Thammarat Aree, Chalisa Sabphon, Pattara Sawasdee

**Affiliations:** a Department of Chemistry, Faculty of Science, Chulalongkorn University, Phyathai Road, Pathumwan, Bangkok 10330, Thailand; b The Center for Petroleum, Petrochemicals, and Advanced Materials, Chulalongkorn University, Bangkok 10330, Thailand

## Abstract

In the title compound (alternatively called 4′,5,7-trimethoxy­flavone methanol solvate hydrate), C_18_H_16_O_5_·CH_3_OH·H_2_O, the flavone mol­ecule is almost planar, the inter­planar angle between the planes of the benzopyran-4-one group and the attached benzene ring being 4.69 (9)°. In the crystal, the flavone mol­ecule makes inter­molecular C—H⋯O hydrogen bonds to adjacent inversion-related flavone mol­ecules, generating *R*_2_^2^(8) and *R*_2_^2^(14) rings and an infinite ribbon. The inversion-related ribbons are stabilized through the inter­stitial water and methanol mol­ecules *via* inter­molecular O—H⋯O hydrogen bonds, generating *R*_4_^2^(8) and *R*_2_^1^(6) rings and *C*_2_^2^(4) chains, and are further sustained by π–π inter­actions with an inter­planar spacing of 3.365 (2)Å.

## Related literature

For related structures, see: Teh *et al.* (2005 ▶) and the Cambridge Structural Database (Allen, 2002 ▶). For the graph-set description of hydrogen-bond patterns, see: Bernstein *et al.* (1995 ▶). For *CONQUEST*, see: Bruno *et al.* (2002 ▶).
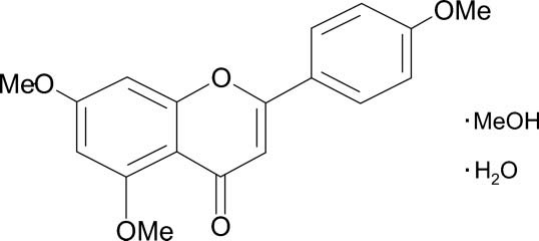


## Experimental

### 

#### Crystal data

C_18_H_16_O_5_·CH_4_O·H_2_O
*M* *_r_* = 362.37
Triclinic, 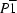

*a* = 9.5333 (2) Å
*b* = 9.8861 (3) Å
*c* = 10.5378 (3) Å
α = 86.671 (1)°
β = 66.101 (1)°
γ = 78.488 (1)°
*V* = 889.45 (4) Å^3^
*Z* = 2
Mo *K*α radiation
μ = 0.10 mm^−1^
*T* = 298 K
0.48 × 0.46 × 0.28 mm

#### Data collection

Bruker SMART APEXII CCD area-detector diffractometer
Absorption correction: multi-scan (**SADABS**; Bruker, 2005 ▶) *T*_min_ = 0.845, *T*_max_ = 0.916
10931 measured reflections
5238 independent reflections
3026 reflections with *I* > 2σ(*I*)
*R*_int_ = 0.027

#### Refinement

*R*[*F* ^2^ > 2σ(*F* ^2^)] = 0.052
*wR*(*F* ^2^) = 0.176
*S* = 1.04
5238 reflections
248 parameters
H atoms treated by a mixture of independent and constrained refinement
Δρ_max_ = 0.29 e Å^−3^
Δρ_min_ = −0.19 e Å^−3^

### 

Data collection: *APEX2* (Bruker, 2005 ▶); cell refinement: *SAINT* (Bruker, 2005 ▶); data reduction: *SAINT*; program(s) used to solve structure: *SHELXTL* (Sheldrick, 2008 ▶); program(s) used to refine structure: *SHELXTL*; molecular graphics: *ORTEP-3 for Windows* (Farrugia, 1997 ▶) and *Mercury* (Macrae *et al.* 2006 ▶).; software used to prepare material for publication: *SHELXTL*.

## Supplementary Material

Crystal structure: contains datablocks I, global. DOI: 10.1107/S1600536809040513/jh2106sup1.cif

Structure factors: contains datablocks I. DOI: 10.1107/S1600536809040513/jh2106Isup2.hkl

Additional supplementary materials:  crystallographic information; 3D view; checkCIF report

## Figures and Tables

**Table 1:** Hydrogen-bond geometry (Å, °)

*D*—H⋯*A*	*D*—H	H⋯*A*	*D*⋯*A*	*D*—H⋯*A*
O1*W*1—H1*W*1⋯O2^i^	0.80 (3)	2.06 (3)	2.844 (2)	169 (3)
O1*W*1—H2*W*1⋯O2	0.85 (3)	2.15 (3)	2.940 (2)	154 (3)
O1*W*1—H2*W*1⋯O3	0.85 (3)	2.45 (3)	3.113 (2)	134 (3)
O1*M*1—H4*M*1⋯O1*W*1	0.82	2.01	2.822 (3)	173
C14—H14⋯O5^ii^	0.93	2.50	3.418 (2)	168
C17—H17*C*⋯O4^iii^	0.96	2.81	3.287 (2)	112

Symmetry codes: (i) 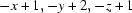; (ii) 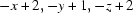; (iii) 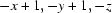.

**Fig. 1.**
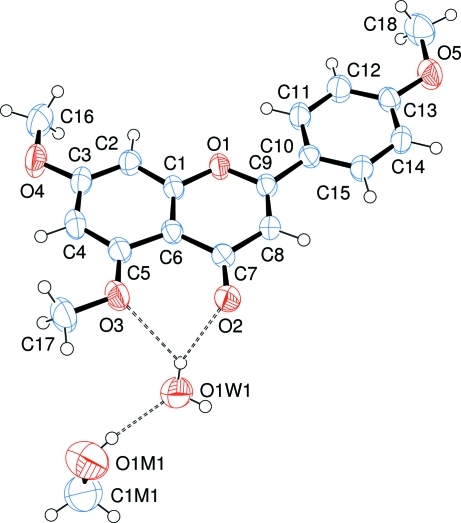
: The structure of (I) with atom numbering and 50% probability displacement ellipsoids. Hydrogen bonds are shown as dashed lines.

**Fig. 2.**
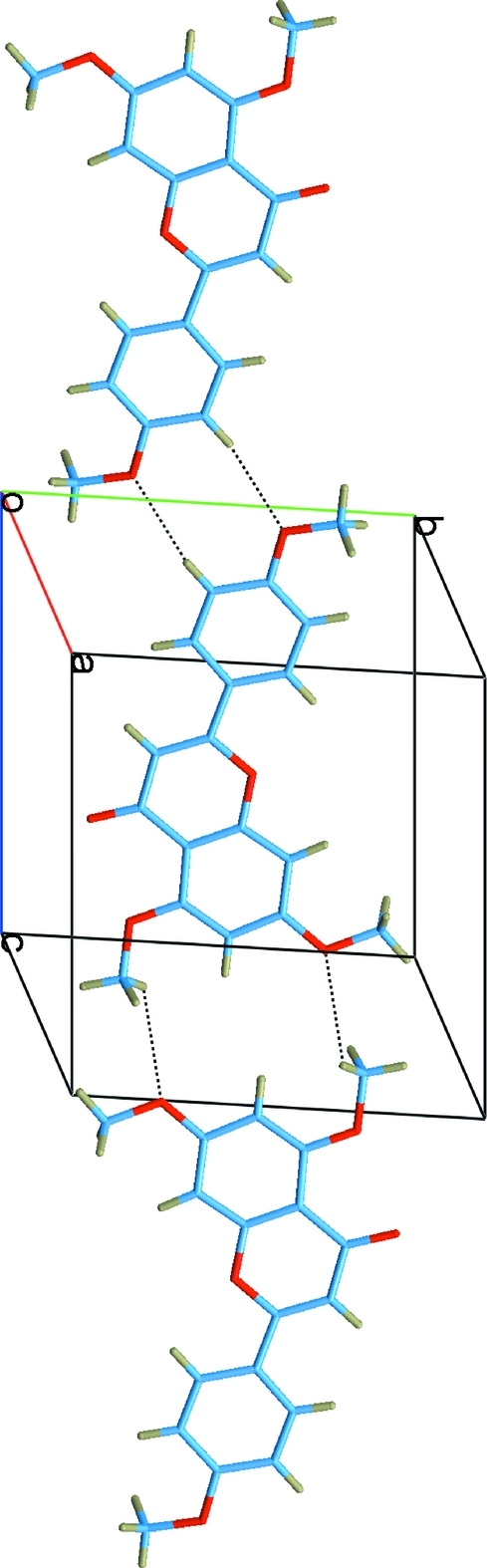
: An infinite ribbon formed by the inversion-related flavone molecules that are making C—H···O hydrogen bonds with ring motifs of R22(8) and R22(14). Hydrogen bonds are shown as dashed lines.

**Fig. 3.**
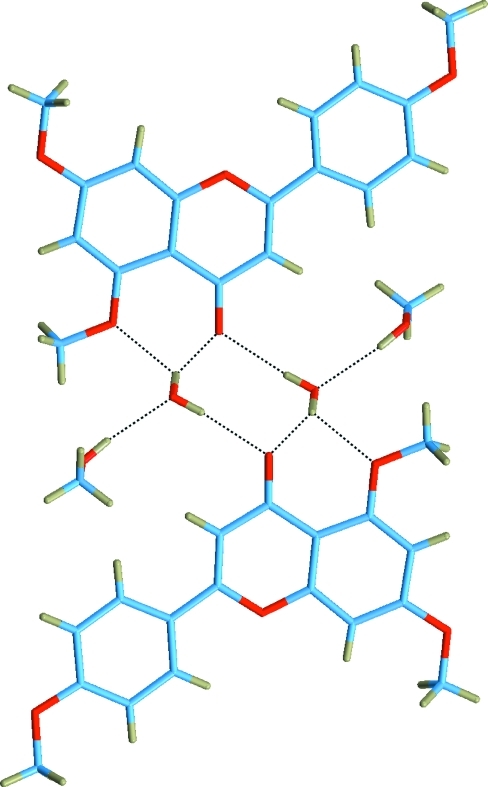
: R42(8), R21(6) rings and C22(4) chains generated from the two inversion-related flavone-methanol-hydrate molecules through O—H···O hydrogen bonds. Hydrogen bonds are shown as dashed lines.

**Fig. 4.**
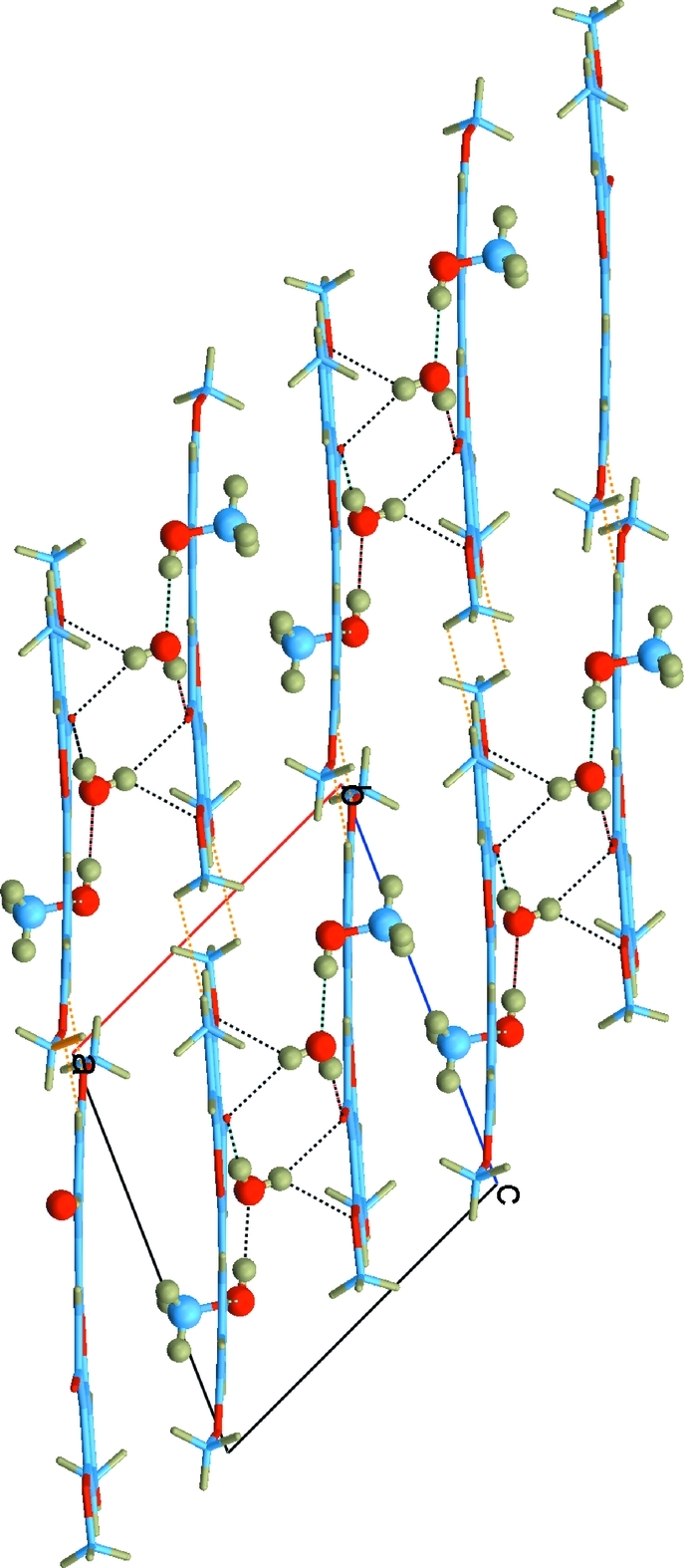
: Parellel, infinite ribbons maintained by intermolecular C—H···O, O—H···O hydrogen bonds and π–π interactions. Hydrogen bonds are shown as dashed lines.

## supplementary crystallographic information

### Comment

The title compound, (I), (4',5,7-trimethoxy-2-phenyl-4*H*-1-benzopyran -4-one or 4',5,7-trimethoxyflavone methanol solvate hydrate), C_18_H_16_O_5_.CH_3_OH.H_2_O (Fig.1), is a secondary metabolite that was isolated from a Thai medicinal plant, *Kaempferia parviflora*. Several flavones have also been isolated from the same plant and their crystal structures have been reported, see: Teh *et al.* (2005) and references cited therein. Here we report another crystal structure of flavone; water and methanol molecules in the interstices play a key role as hydrogen bonding mediator in stabilizing the entire crystal.

The molecular structure of (I) is almost planar; the interplanar angle between the benzopyran-4-one group and the attached phenyl group is 4.69 (9)° (Fig. 1) This observation is consistent with other flavone structures in the Cambridge Structural Database [Version 1.11 (Allen, 2002); CONQUEST (Bruno *et al.*, 2002)]. The three methoxy C-atoms deviate from the mean planes of the two phenyl rings by -0.091 (3), 0.006 (3) and 0.277 (4) Å for atoms C16, C17 and C18, respectively. The corresponding values of torsion angles are: 3.35 (25)°, C16—O4—C3—C2; -2.95 (24)°, C17—O3—C5—C4 and 9.55 (27)°, C18—O5—C13—C12.

In the crystal lattice, the flavone molecule inclines 48.44 (4)° against the *a*-*b* plane and makes intermolecular C—H···O hydrogen bonds to the adjacent inversion-related flavone molecules, generating *R*_2_^2^(8), *R*_2_^2^(14) rings (Bernstein *et al.*, 1995) and an infinite ribbon along the *c* axis (Fig. 2). The inversion-related ribbons are stabilized through the interstitial water and methanol molecules *via* intermolecular O—H···O hydrogen bonds, generating *R*_4_^2^(8), *R*_2_^1^(6) rings and *C*_2_^2^(4) chains (Bernstein *et al.*, 1995) and are further sustained by π–π interactions with an interplanar spacing of 3.365 (2) Å (Figs. 3 and 4).

### Experimental

The title compound, (I), was extracted from *Kaempferia parviflora*, a medicinal plant from the north-east of Thailand. Single crystals of (I) were obtained from slow evaporation of a methanol-water (1:1, *v*/*v*) solution at room temperature. Because the crystals desolvate very easily, the chosen crystal must be soaked in parafin oil prior to mounting on the tip of a glass fiber.

### Refinement

The water H-atoms were located in a difference electron density map and refined isotropically. All other H atoms were located and then refined using a riding model: C—H = 0.93 Å (aromatic), *U*_iso_(H) = 1.2*U*_eq_(C) and C—H = 0.96 Å (methyl), O—H = 0.82 Å (hydroxyl), *U*_iso_(H) = 1.5*U*_eq_(C/O).

### Crystal data

**Table:**

C_18_H_16_O_5_·CH_4_O·H_2_O	*Z* = 2
*M**_r_* = 362.37	*F*(000) = 384
Triclinic, *P*1	*D*_x_ = 1.353 Mg m^−^^3^
Hall symbol: -P 1	Melting point: not measured K
*a* = 9.5333 (2) Å	Mo *K*α radiation, λ = 0.71073 Å
*b* = 9.8861 (3) Å	Cell parameters from 3400 reflections
*c* = 10.5378 (3) Å	θ = 2.5–30.1°
α = 86.671 (1)°	µ = 0.10 mm^−^^1^
β = 66.101 (1)°	*T* = 298 K
γ = 78.488 (1)°	Block, colourless
*V* = 889.45 (4) Å^3^	0.48 × 0.46 × 0.28 mm

### Data collection

**Table:**

Bruker SMART APEXII CCD area-detector diffractometer	5238 independent reflections
Radiation source: fine-focus sealed tube	3026 reflections with *I* > 2σ(*I*)
graphite	*R*_int_ = 0.027
φ and ω scans	θ_max_ = 30.5°, θ_min_ = 2.1°
Absorption correction: multi-scan (*SADABS*; Bruker, 2005)	*h* = −9→13
*T*_min_ = 0.845, *T*_max_ = 0.916	*k* = −13→14
10931 measured reflections	*l* = −14→14

### Refinement

**Table:**

Refinement on *F*^2^	Primary atom site location: structure-invariant direct methods
Least-squares matrix: full	Secondary atom site location: difference Fourier map
*R*[*F*^2^ > 2σ(*F*^2^)] = 0.052	Hydrogen site location: inferred from neighbouring sites
*wR*(*F*^2^) = 0.176	H atoms treated by a mixture of independent and constrained refinement
*S* = 1.03	*w* = 1/[σ^2^(*F*_o_^2^) + (0.0831*P*)^2^ + 0.1044*P*] where *P* = (*F*_o_^2^ + 2*F*_c_^2^)/3
5238 reflections	(Δ/σ)_max_ < 0.001
248 parameters	Δρ_max_ = 0.28 e Å^−^^3^
0 restraints	Δρ_min_ = −0.19 e Å^−^^3^

### Special details

**Table:**

Geometry. All e.s.d.'s (except the e.s.d. in the dihedral angle between two l.s. planes) are estimated using the full covariance matrix. The cell e.s.d.'s are taken into account individually in the estimation of e.s.d.'s in distances, angles and torsion angles; correlations between e.s.d.'s in cell parameters are only used when they are defined by crystal symmetry. An approximate (isotropic) treatment of cell e.s.d.'s is used for estimating e.s.d.'s involving l.s. planes.
Refinement. Refinement of *F*^2^ against ALL reflections. The weighted *R*-factor *wR* and goodness of fit *S* are based on *F*^2^, conventional *R*-factors *R* are based on *F*, with *F* set to zero for negative *F*^2^. The threshold expression of *F*^2^ > σ(*F*^2^) is used only for calculating *R*-factors(gt) *etc*. and is not relevant to the choice of reflections for refinement. *R*-factors based on *F*^2^ are statistically about twice as large as those based on *F*, and *R*- factors based on ALL data will be even larger.

### Fractional atomic coordinates and isotropic or equivalent isotropic displacement parameters (Å^2^)

**Table:**

	*x*	*y*	*z*	*U*_iso_*/*U*_eq_	
C1	0.68788 (16)	0.47269 (15)	0.38254 (14)	0.0358 (3)	
C2	0.66882 (17)	0.35992 (16)	0.32291 (16)	0.0412 (3)	
H2	0.6873	0.2711	0.3544	0.049*	
C3	0.62134 (18)	0.38517 (17)	0.21510 (16)	0.0440 (4)	
C4	0.59294 (19)	0.51854 (18)	0.16889 (16)	0.0464 (4)	
H4	0.5621	0.5331	0.0952	0.056*	
C5	0.61000 (17)	0.62860 (16)	0.23116 (16)	0.0414 (4)	
C6	0.65977 (16)	0.60841 (15)	0.34281 (14)	0.0365 (3)	
C7	0.68386 (17)	0.71706 (16)	0.41605 (16)	0.0397 (3)	
C8	0.74295 (17)	0.66940 (15)	0.51967 (16)	0.0398 (3)	
H8	0.7663	0.7337	0.5654	0.048*	
C9	0.76598 (16)	0.53622 (15)	0.55337 (15)	0.0357 (3)	
C10	0.82311 (16)	0.47626 (15)	0.65810 (15)	0.0367 (3)	
C11	0.8392 (2)	0.33617 (17)	0.68317 (18)	0.0481 (4)	
H11	0.8101	0.2794	0.6346	0.058*	
C12	0.8978 (2)	0.27859 (17)	0.77914 (19)	0.0524 (4)	
H12	0.9068	0.1844	0.7953	0.063*	
C13	0.94259 (18)	0.36194 (17)	0.85025 (16)	0.0435 (4)	
C14	0.9241 (2)	0.50237 (17)	0.82922 (18)	0.0502 (4)	
H14	0.9519	0.5589	0.8790	0.060*	
C15	0.8645 (2)	0.55897 (16)	0.73453 (17)	0.0471 (4)	
H15	0.8517	0.6538	0.7216	0.056*	
C16	0.6139 (3)	0.1483 (2)	0.1950 (2)	0.0746 (6)	
H16A	0.7186	0.1175	0.1887	0.112*	
H16B	0.5930	0.0887	0.1386	0.112*	
H16C	0.5413	0.1459	0.2898	0.112*	
C17	0.5388 (3)	0.7859 (2)	0.0770 (2)	0.0683 (6)	
H17A	0.6217	0.7394	−0.0043	0.102*	
H17B	0.5208	0.8833	0.0617	0.102*	
H17C	0.4451	0.7515	0.0960	0.102*	
C18	1.0500 (3)	0.1713 (2)	0.9532 (2)	0.0734 (6)	
H18A	0.9585	0.1306	0.9865	0.110*	
H18B	1.1006	0.1529	1.0165	0.110*	
H18C	1.1206	0.1324	0.8633	0.110*	
O1	0.73689 (12)	0.43770 (10)	0.48755 (11)	0.0407 (3)	
O2	0.65502 (15)	0.84261 (11)	0.39405 (13)	0.0565 (3)	
O3	0.58166 (15)	0.76114 (12)	0.19255 (13)	0.0564 (3)	
O5	1.00623 (17)	0.31561 (13)	0.94348 (14)	0.0645 (4)	
O4	0.59709 (17)	0.28528 (13)	0.14734 (14)	0.0622 (4)	
O1W1	0.3293 (2)	0.95361 (18)	0.4350 (2)	0.0783 (5)	
H1W1	0.326 (3)	1.006 (3)	0.491 (3)	0.085 (9)*	
H2W1	0.423 (4)	0.909 (3)	0.401 (3)	0.118 (11)*	
C1M1	0.0302 (3)	1.0928 (3)	0.3320 (3)	0.0925 (8)	
H1M1	−0.0014	1.0107	0.3785	0.139*	
H2M1	−0.0245	1.1223	0.2732	0.139*	
H3M1	0.0059	1.1644	0.3997	0.139*	
O1M1	0.1882 (2)	1.0657 (2)	0.2530 (2)	0.1045 (6)	
H4M1	0.2341	1.0277	0.3005	0.157*	

### Atomic displacement parameters (Å^2^)

**Table:**

	*U*^11^	*U*^22^	*U*^33^	*U*^12^	*U*^13^	*U*^23^
C1	0.0318 (7)	0.0458 (8)	0.0319 (7)	−0.0084 (6)	−0.0146 (6)	−0.0004 (6)
C2	0.0418 (8)	0.0431 (8)	0.0430 (8)	−0.0107 (6)	−0.0197 (7)	−0.0018 (6)
C3	0.0442 (8)	0.0532 (9)	0.0404 (8)	−0.0151 (7)	−0.0193 (7)	−0.0059 (7)
C4	0.0491 (9)	0.0617 (10)	0.0359 (8)	−0.0135 (7)	−0.0232 (7)	0.0005 (7)
C5	0.0395 (8)	0.0489 (9)	0.0384 (8)	−0.0070 (6)	−0.0190 (6)	0.0009 (6)
C6	0.0314 (7)	0.0451 (8)	0.0343 (7)	−0.0069 (6)	−0.0144 (6)	−0.0015 (6)
C7	0.0379 (8)	0.0428 (8)	0.0401 (8)	−0.0036 (6)	−0.0191 (6)	−0.0017 (6)
C8	0.0427 (8)	0.0399 (8)	0.0431 (8)	−0.0052 (6)	−0.0240 (7)	−0.0055 (6)
C9	0.0306 (7)	0.0436 (8)	0.0349 (7)	−0.0074 (6)	−0.0146 (6)	−0.0032 (6)
C10	0.0325 (7)	0.0428 (8)	0.0363 (8)	−0.0062 (6)	−0.0157 (6)	−0.0011 (6)
C11	0.0601 (10)	0.0449 (9)	0.0531 (10)	−0.0139 (7)	−0.0349 (8)	0.0000 (7)
C12	0.0710 (12)	0.0409 (8)	0.0579 (11)	−0.0122 (8)	−0.0388 (9)	0.0070 (7)
C13	0.0454 (8)	0.0506 (9)	0.0391 (8)	−0.0068 (7)	−0.0230 (7)	0.0023 (7)
C14	0.0647 (11)	0.0472 (9)	0.0528 (10)	−0.0103 (8)	−0.0372 (9)	−0.0047 (7)
C15	0.0599 (10)	0.0396 (8)	0.0518 (10)	−0.0074 (7)	−0.0335 (8)	−0.0005 (7)
C16	0.1022 (17)	0.0561 (12)	0.0855 (16)	−0.0196 (11)	−0.0542 (14)	−0.0117 (11)
C17	0.0953 (16)	0.0674 (12)	0.0657 (12)	−0.0162 (11)	−0.0574 (12)	0.0121 (10)
C18	0.0943 (16)	0.0652 (13)	0.0775 (14)	−0.0155 (11)	−0.0544 (13)	0.0231 (11)
O1	0.0474 (6)	0.0425 (6)	0.0420 (6)	−0.0110 (5)	−0.0268 (5)	0.0009 (4)
O2	0.0769 (8)	0.0409 (6)	0.0661 (8)	−0.0021 (6)	−0.0472 (7)	−0.0007 (5)
O3	0.0778 (8)	0.0520 (7)	0.0570 (8)	−0.0099 (6)	−0.0468 (7)	0.0064 (6)
O5	0.0891 (10)	0.0608 (8)	0.0630 (8)	−0.0100 (7)	−0.0531 (8)	0.0069 (6)
O4	0.0881 (9)	0.0601 (8)	0.0603 (8)	−0.0243 (7)	−0.0464 (7)	−0.0048 (6)
O1W1	0.0728 (11)	0.0668 (10)	0.1064 (14)	−0.0040 (8)	−0.0491 (10)	−0.0164 (9)
C1M1	0.0895 (18)	0.0874 (18)	0.101 (2)	−0.0153 (14)	−0.0391 (16)	−0.0007 (15)
O1M1	0.0944 (13)	0.1108 (15)	0.0973 (14)	−0.0054 (11)	−0.0355 (11)	0.0138 (11)

### Geometric parameters (Å, °)

**Table:**

C1—O1	1.3683 (16)	C13—O5	1.3649 (18)
C1—C2	1.388 (2)	C13—C14	1.380 (2)
C1—C6	1.389 (2)	C14—C15	1.378 (2)
C2—C3	1.377 (2)	C14—H14	0.9300
C2—H2	0.9300	C15—H15	0.9300
C3—O4	1.3584 (18)	C16—O4	1.418 (2)
C3—C4	1.392 (2)	C16—H16A	0.9600
C4—C5	1.372 (2)	C16—H16B	0.9600
C4—H4	0.9300	C16—H16C	0.9600
C5—O3	1.3570 (19)	C17—O3	1.427 (2)
C5—C6	1.427 (2)	C17—H17A	0.9600
C6—C7	1.463 (2)	C17—H17B	0.9600
C7—O2	1.2458 (18)	C17—H17C	0.9600
C7—C8	1.437 (2)	C18—O5	1.414 (2)
C8—C9	1.342 (2)	C18—H18A	0.9600
C8—H8	0.9300	C18—H18B	0.9600
C9—O1	1.3597 (16)	C18—H18C	0.9600
C9—C10	1.4656 (19)	O1W1—H1W1	0.80 (3)
C10—C11	1.385 (2)	O1W1—H2W1	0.85 (3)
C10—C15	1.391 (2)	C1M1—O1M1	1.373 (3)
C11—C12	1.387 (2)	C1M1—H1M1	0.9600
C11—H11	0.9300	C1M1—H2M1	0.9600
C12—C13	1.376 (2)	C1M1—H3M1	0.9600
C12—H12	0.9300	O1M1—H4M1	0.8200
			
O1—C1—C2	113.11 (13)	C12—C13—C14	120.00 (14)
O1—C1—C6	122.23 (12)	C15—C14—C13	120.15 (14)
C2—C1—C6	124.65 (13)	C15—C14—H14	119.9
C3—C2—C1	117.17 (14)	C13—C14—H14	119.9
C3—C2—H2	121.4	C14—C15—C10	120.92 (15)
C1—C2—H2	121.4	C14—C15—H15	119.5
O4—C3—C2	123.74 (15)	C10—C15—H15	119.5
O4—C3—C4	115.09 (13)	O4—C16—H16A	109.5
C2—C3—C4	121.16 (13)	O4—C16—H16B	109.5
C5—C4—C3	120.54 (14)	H16A—C16—H16B	109.5
C5—C4—H4	119.7	O4—C16—H16C	109.5
C3—C4—H4	119.7	H16A—C16—H16C	109.5
O3—C5—C4	123.39 (13)	H16B—C16—H16C	109.5
O3—C5—C6	115.92 (13)	O3—C17—H17A	109.5
C4—C5—C6	120.69 (14)	O3—C17—H17B	109.5
C1—C6—C5	115.76 (12)	H17A—C17—H17B	109.5
C1—C6—C7	118.57 (12)	O3—C17—H17C	109.5
C5—C6—C7	125.67 (13)	H17A—C17—H17C	109.5
O2—C7—C8	120.81 (13)	H17B—C17—H17C	109.5
O2—C7—C6	124.13 (13)	O5—C18—H18A	109.5
C8—C7—C6	115.05 (13)	O5—C18—H18B	109.5
C9—C8—C7	122.92 (13)	H18A—C18—H18B	109.5
C9—C8—H8	118.5	O5—C18—H18C	109.5
C7—C8—H8	118.5	H18A—C18—H18C	109.5
C8—C9—O1	120.88 (12)	H18B—C18—H18C	109.5
C8—C9—C10	127.70 (12)	C9—O1—C1	120.21 (11)
O1—C9—C10	111.42 (12)	C5—O3—C17	117.97 (13)
C11—C10—C15	117.96 (13)	C13—O5—C18	117.91 (13)
C11—C10—C9	121.38 (12)	C3—O4—C16	118.09 (14)
C15—C10—C9	120.66 (13)	H1W1—O1W1—H2W1	105 (3)
C10—C11—C12	121.46 (14)	O1M1—C1M1—H1M1	109.5
C10—C11—H11	119.3	O1M1—C1M1—H2M1	109.5
C12—C11—H11	119.3	H1M1—C1M1—H2M1	109.5
C13—C12—C11	119.46 (15)	O1M1—C1M1—H3M1	109.5
C13—C12—H12	120.3	H1M1—C1M1—H3M1	109.5
C11—C12—H12	120.3	H2M1—C1M1—H3M1	109.5
O5—C13—C12	124.34 (15)	C1M1—O1M1—H4M1	109.5
O5—C13—C14	115.66 (13)		
			
O1—C1—C2—C3	−179.29 (13)	C8—C9—C10—C11	179.11 (15)
C6—C1—C2—C3	1.2 (2)	O1—C9—C10—C11	−1.3 (2)
C1—C2—C3—O4	−179.64 (15)	C8—C9—C10—C15	−1.6 (2)
C1—C2—C3—C4	−0.4 (2)	O1—C9—C10—C15	178.04 (13)
O4—C3—C4—C5	178.57 (15)	C15—C10—C11—C12	−1.4 (2)
C2—C3—C4—C5	−0.8 (2)	C9—C10—C11—C12	177.97 (15)
C3—C4—C5—O3	−178.88 (14)	C10—C11—C12—C13	−0.7 (3)
C3—C4—C5—C6	1.1 (2)	C11—C12—C13—O5	−177.82 (16)
O1—C1—C6—C5	179.68 (13)	C11—C12—C13—C14	2.2 (3)
C2—C1—C6—C5	−0.9 (2)	O5—C13—C14—C15	178.39 (15)
O1—C1—C6—C7	0.1 (2)	C12—C13—C14—C15	−1.6 (3)
C2—C1—C6—C7	179.53 (14)	C13—C14—C15—C10	−0.5 (3)
O3—C5—C6—C1	179.67 (13)	C11—C10—C15—C14	1.9 (2)
C4—C5—C6—C1	−0.3 (2)	C9—C10—C15—C14	−177.40 (14)
O3—C5—C6—C7	−0.8 (2)	C8—C9—O1—C1	2.0 (2)
C4—C5—C6—C7	179.24 (14)	C10—C9—O1—C1	−177.67 (11)
C1—C6—C7—O2	−176.29 (14)	C2—C1—O1—C9	177.81 (12)
C5—C6—C7—O2	4.2 (2)	C6—C1—O1—C9	−2.7 (2)
C1—C6—C7—C8	3.0 (2)	C4—C5—O3—C17	−2.9 (2)
C5—C6—C7—C8	−176.58 (14)	C6—C5—O3—C17	177.07 (15)
O2—C7—C8—C9	175.49 (15)	C12—C13—O5—C18	9.6 (3)
C6—C7—C8—C9	−3.8 (2)	C14—C13—O5—C18	−170.45 (18)
C7—C8—C9—O1	1.4 (2)	C2—C3—O4—C16	3.4 (3)
C7—C8—C9—C10	−179.01 (14)	C4—C3—O4—C16	−175.96 (16)

### Hydrogen-bond geometry (Å, °)

**Table:**

*D*—H···*A*	*D*—H	H···*A*	*D*···*A*	*D*—H···*A*
O1W1—H1W1···O2^i^	0.80 (3)	2.06 (3)	2.844 (2)	169 (3)
O1W1—H2W1···O2	0.85 (3)	2.15 (3)	2.940 (2)	154 (3)
O1W1—H2W1···O3	0.85 (3)	2.45 (3)	3.113 (2)	134 (3)
O1M1—H4M1···O1W1	0.82	2.01	2.822 (3)	173
C14—H14···O5^ii^	0.93	2.50	3.418 (2)	168
C17—H17C···O4^iii^	0.96	2.81	3.287 (2)	112

Symmetry codes: (i) −*x*+1, −*y*+2, −*z*+1; (ii) −*x*+2, −*y*+1, −*z*+2; (iii) −*x*+1, −*y*+1, −*z*.
